# Supplementary material for: Impact of Reduced Image Noise on Deauville Scores in Patients with Lymphoma Scanned on a Long-Axial Field-of-View PET/CT-Scanner
Source: Diagnostics (Basel). 2023 Mar 2;13(5):947. doi: 10.3390/diagnostics13050947 (PMC10000539; doi:10.3390/diagnostics13050947)
Supplement: Supplementary file 1 [file diagnostics-13-00947-s001.zip › Figure S2a+b.pdf]

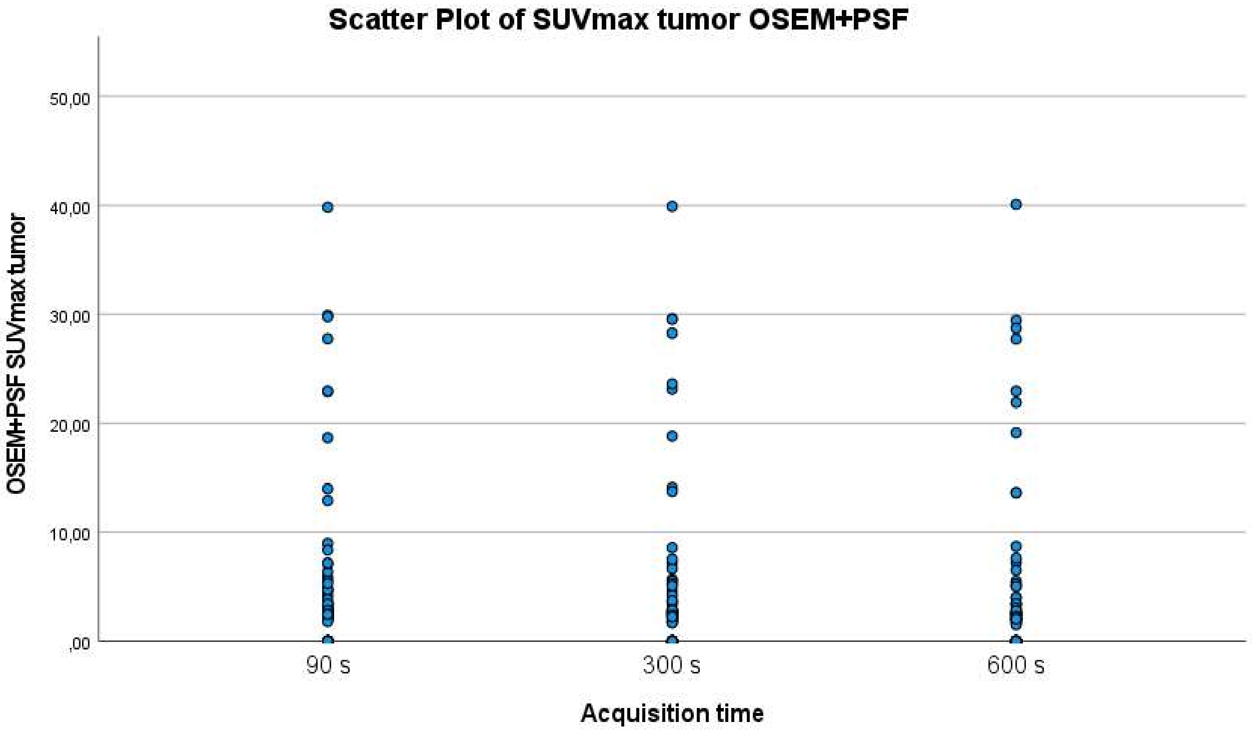

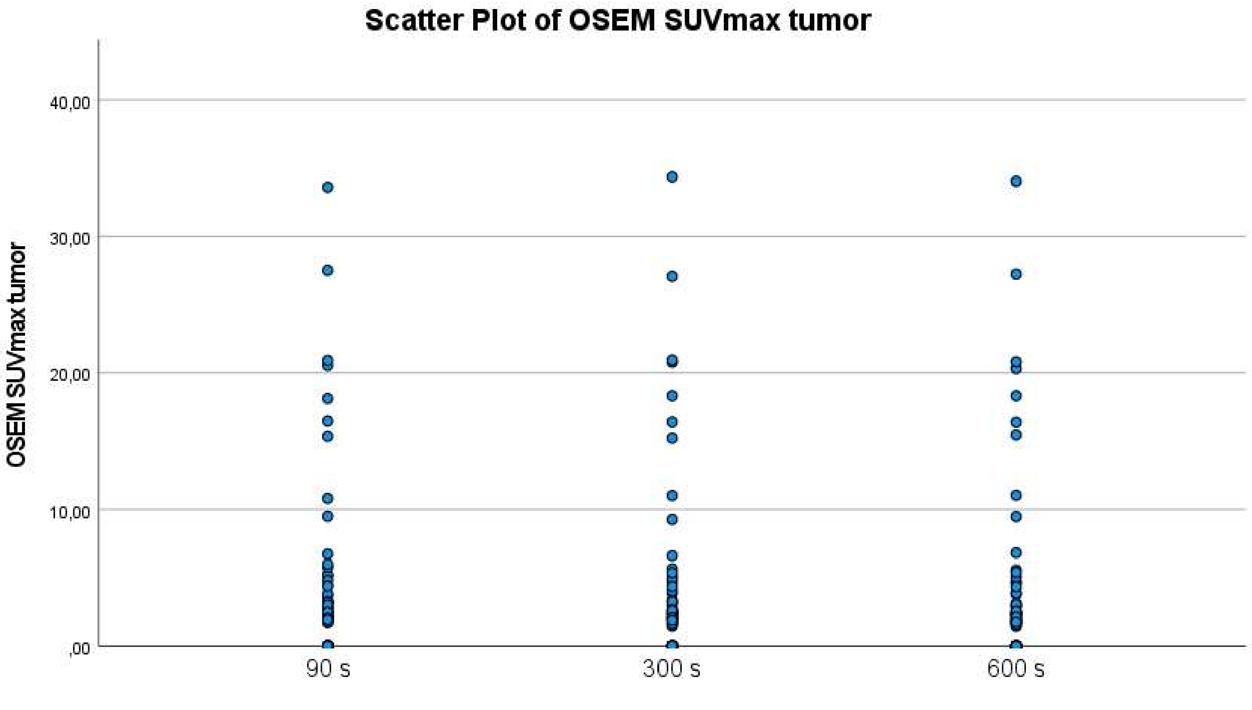

Figure S2a+b: Tumor SUVmax for OSEM+PSF and OSEM only, no significant change in SUVmax in tumor are seen between acquisition times.
