# Supplementary material for: Impact of Reduced Image Noise on Deauville Scores in Patients with Lymphoma Scanned on a Long-Axial Field-of-View PET/CT-Scanner
Source: Diagnostics (Basel). 2023 Mar 2;13(5):947. doi: 10.3390/diagnostics13050947 (PMC10000539; doi:10.3390/diagnostics13050947)
Supplement: Supplementary file 1 [file diagnostics-13-00947-s001.zip › Figure S4.pdf]

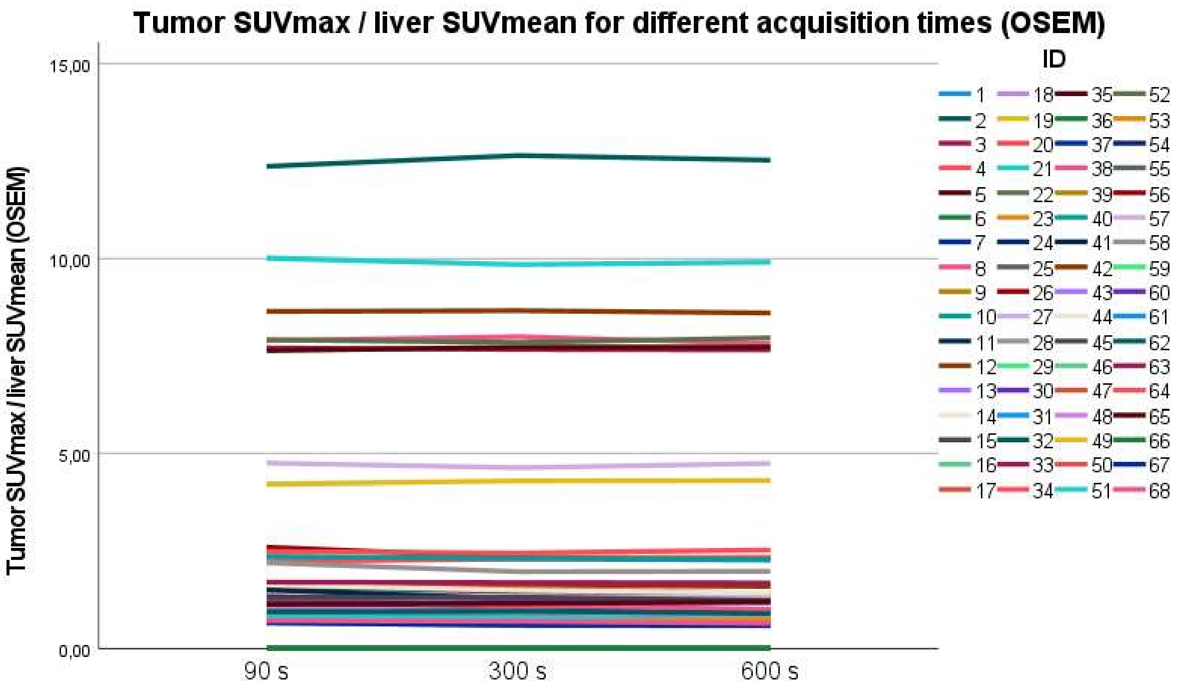

Figure S4: **Tumor SUVmax/liver SUVmean ratio remains stable with increasing acquisition time for OSEM**
